# Stimulation of Pro-inflammatory Cytokines in Mixed Cultures of Peripheral Blood Mononuclear Cells and Anaerobic Bacteria

**DOI:** 10.7759/cureus.50586

**Published:** 2023-12-15

**Authors:** Anthony R Torres, Shayne Morris, Michael Benson, Craig Wilkinson, Rachael Lyon

**Affiliations:** 1 Microbiology, Nutri-Biome, Ogden, USA; 2 Statistics, Nutri-Biome, Ogden, USA; 3 Consultancy, Nutri-Biome, Ogden, USA

**Keywords:** inflammation, chemiluminescence immunoassay, human microbiome, proinflammatory cytokines, anaerobic bacteria, peripheral blood mononuclear cells (pbmc)

## Abstract

In the last couple of decades, much progress has been made in studying bacteria living in humans. However, there is much more to learn about bacteria immune cell interactions. Here, we show that anaerobic bacteria do not grow when cultured overnight with human cells under atmospheric air. Air contains about 18% oxygen, which inhibits the growth of these bacteria while supporting the cultivation of human cells. The bacteria cultured with human peripheral blood mononuclear cells (PBMCs) inflamed with phytohemagglutinin (PHA) greatly increased the production of proinflammatory cytokines like tumor necrosis factor-alpha (TNFα) while inhibiting the production of monocyte chemoattractant protein-1 (MCP-1), an important chemokine.

## Introduction

The symbiotic relationship between host and microorganisms is extremely complicated, and it is important to better understand these cellular interactions [1]. Humans are colonized by the microbiota containing bacteria, archaea, fungi, and viruses; different body surfaces contain distinct members. Internal organs, such as the heart, once thought to be sterile, harbor organ-specific microorganisms [2]. Blood from healthy individuals has diverse microbiota [3], and specific bacteria even exist in immune cells [4]. The colon is home to the most diverse microbiota composed of 100 trillion bacteria containing about 25 times the number of genes as the Homo sapiens genome [5]. The gut microbiota plays an important role in crosstalk between gut microbes and host cells as microbial metabolites can cross the intestinal wall and enter the bloodstream. Over 200 microbial metabolites have been identified in local and distal systems of the body [6]. The gut is also home to about 70-80% of human immune cells [7], and the symbiotic relationships between the microbiota and cells of the immune system are important in numerous medical disorders [8].

It is important to better understand how bacteria and immune cells coexist in the gut. It is commonly assumed that mixing bacteria with human cells in culture would result in the bacteria overgrowing the cells. We assumed that the oxygen in cell culture media would inhibit the growth or kill the anaerobic bacteria, allowing cellular reactions to be measured. Experiments adding a commercial mixture of probiotic anaerobic bacteria to peripheral blood mononuclear cells (PBMC) in culture with atmosphere air, which contains about 18% oxygen, did not show gross signs of bacterial contamination, and the growth rates were similar to control cultures at 24 hours. Here, we demonstrate that the four probiotics induced small amounts of cytokines/chemokines in PBMC, and cultures containing phytohemagglutinin (PHA) showed higher cytokine concentrations. There is a lot to learn from these mixed cultures, as the combination of PHA and probiotic bacteria resulted in significantly higher concentrations of the pro-inflammatory cytokine IL-1β, whereas the combination of PHA and bacteria significantly decreased the production of the chemokine MCP-1.

## Materials and methods

Study design

The study involved four commercial anaerobic probiotic bacterial products: Neuro Byome (NB), Metabo Byome (MB), Male/Female Byome (M/F), and Immuno Byome (IB) were obtained from Nutri-Biome (Ogden, Utah). The four probiotics were made by reconstituting 18 freeze-dried (Gram - and Gram +) anaerobic bacteria and growing them on individual agar plates (Table 1). The agar plates with bacteria were incubated under strict anaerobic conditions (90% nitrogen, 5% carbon dioxide, 5% hydrogen at 37 °C) in a Bactron EZ Anaerobic Chamber (Sheldon Manufacturing, Cornelius, OR, USA).

**Table 1:** Anaerobic bacteria strains in four probiotic products Gram + and gram- are listed as + or -. The particular agar used to grow the specific strain is shown in the second column.

Neuro Byome (NB)	Agar
Faecalibacterium duncaniae (+)	Wilkins-Chalgren Agar
Bacteroides fragilis (-)	Tryptic Soy Agar + 5% Sheep Blood
Parabacteroides distasonis (-)	Wilkins-Chalgren Agar
Agathobaculum butyriciproducens (+)	Wilkins-Chalgren Agar
Metabo Byome (MB)	
Akkermansia muciniphila (-)	Brain Heart Infusion Agar + 0.3% Porcine Mucin (Type II)
Eubacterium rectale (+)	Wilkins-Chalgren Agar
Butyricicoccus pullicaecorum (+)	Wilkins-Chalgren Agar
Anaerobutyricum hallii (+)	Wilkins-Chalgren Agar
Dorea longicatena (+)	Wilkins-Chalgren Agar
Christensenella minuta (-)	Wilkins-Chalgren Agar
Male/Female Byome (MF)	
Eubacterium limosum (+)	Tryptic Soy Agar + 5% Sheep Blood
Bacteroides thetaiotaomicron (-)	Tryptic Soy Agar + 5% Sheep Blood
Bacteroides uniformis (-)	Tryptic Soy Agar + 5% Sheep Blood
Immuno Byome (Immuno)	
Bacteroides ovatus (-)	Tryptic Soy Agar + 5% Sheep Blood
Bacteroides uniformis (-)	Tryptic Soy Agar + 5% Sheep Blood
Clostridium symbiosum (+)	Wilkins-Chalgren Agar
Collinsella aerofaciens (+)	MRS Agar
Roseburia hominis (+)	Wilkins-Chalgren Agar
Anaerostipes caccae (+)	Wilkins-Chalgren Agar

Bacteria were scrapped off the plates after one to five days of incubation depending on the growth of the particular strain. The bacteria were diluted in phosphate-buffered saline (PBS) to obtain an absorbance reading of about 1.0 at 600 nm on an Agilent 453E spectrophotometer (Santa Clara, CA, US). Equal numbers of each bacterium strain were combined to make the four probiotic products contain 4-6X 10^8^ bacteria/ml. Each strain was characterized by DNA typing with strain-specific PCR primers (Table 2).

**Table 2:** Bacterial strain PCR primers Individual bacteria strains were characterized by DNA typing with strain-specific polymerase chain reaction (PCR) primers. The forward and reverse primers are shown in columns 2 and 3, respectively. Published references for the strain-specific primers are shown in column 4.

Strain-Specific PCR Primers (5’--3’)			
Neuro Byome (NB)	Forward	Reverse	Reference
F. duncaniae (+)	TCATCACGCCCAGATTGTCC	GGCGAGTATGTCCAGTTCGT	[9]
B. fragilis (-)	GTACACACCGCCCGT	AATTTAGAACCAATGAACG	[10]
P. distasonis (-)	AATACCGCATGAAGCAGG	GACACGTCCCGCACTTTA	[11]
A. butyriciproducens (+)	GATCACTCTAGCCGGACTGC	GTTAGGCTACGGACTTCGGG	[9]
Metabo Byome (MB)			
A. muciniphila (-)	CAGCACGTGAAGGTGGGGAC	CCTTGCGGTTGGCTTCAGAT	[12]
E. rectale (+)	AAGGGAAGCAAAGCTGTGAA	CGGTTAGGTCACTGGCTTC	[13]
B. pullicaecorum (+)	CGAGCAGGCAAACGACAA	CCAGGTCTTGGTACCGTCC	[14]
A. hallii* (+)	TAATCGGTGCTTTCCTTCG	CAGCCTTACCTGCTGGCTAC	[15]
D. longicatena (+)	CGCATAAGACCACGTACC	TGATAGAAGTTTACATACCGAAAT	[9]
C. minuta (-)	GTAATACGTAGGGAGCAAGC	CCCTCTCCTGTACTCAAGTC	[16]
Male/Female Byome (MF)			
E. limosum (+)	GGCTTGCTGGACAAATACTG	CTAGGCTCGTCAGAGGATG	[17]
B. thetaiotaomicron (-)	AACAGGTGGAAGCTGCGGA	AGCCTCCAACCGCATCAA	[18]
B. uniformis (-)	TATGCAACCAAGCTGATGAACGAAG	AGAGGTTGGCCACGATGTTGATAC	[19]
Immuno Byome (Immuno)			
B. ovatus (-)	GTACACACCGCCCGT	AATATTGCATACTCGAACAC	[9]
B. uniformis (-)	TATGCAACCAAGCTGATGAACGAAG	AGAGGTTGGCCACGATGTTGATAC	[19]
C. symbiosum (+)	GTGAGATGATGTGCCAGGC	TACCGGTTGCTTCGTCGATT	[20]
C. aerofaciens (+)	CCCGACGGGAGGGGAT	CTTCTGCAGGTACAGTCTTGA	[21]
R. hominis (+)	GCACTTTAATTGATTTCTTCG	TCTTAGTCAGGTACCGTCATT	[22]
A. caccae (+)	GTTTTCGGATGGATTTCCTATAT	CTTTTCACACTGAATCATGCGATT	[23]

Human PBMCs from an individual donor were purchased from Cellular Technology Limited (CTL) (Shaker Heights, Ohio, USA). PBMCs contain a mixture of immune cells including T-cells, B-cells, NK cells, monocytes (blood macrophages), and dendritic cells. The complex mixture of immune cells in systemic PBMC makes it possible to study many immune interactions in test tubes. The PBMCs were stored in the vapor phase of liquid nitrogen until the day of use. PBMC culture reagents were obtained from CTL and company protocols were followed. A vial of PBMCs contains about 1 x10^7^ cells, which were plated at 2 x10^5^ cells/well in 200 μL of CTL media in flat-bottom 96-well plates.

Ten microliters containing 4-6 x 10^6^ of the four probiotic bacteria products were added to each well in quadruplicate and allowed to incubate overnight with PBMC cultures under room air containing about 18% oxygen. At 24 hours, there were no signs of contamination or bacterial overgrowth (no cytotoxic granules or anomalous morphologies). To compare the amount of inflammation introduced by these anaerobic bacteria, PBMC control cultures were inflamed with 10 μg/ml PHA. PHA is a well-known mitogen that binds to toll-like receptor 2 (TLR2) on T-cells and monocytes and causes the production of high levels of inflammatory cytokines, which are associated with inflammation [24].

The HIEC-6 normal small intestine epithelial cell line obtained from American Type Culture Collection (ATCC) #CCL-3266 was cultured in Minimum Essential Media (MEM) with Glutamax^TM^ supplemented with 10 ng/ml EGF and 5% fetal bovine serum (FBS). HIEC-6 cells were plated at a density of 10,000 cells per well in 200 μl of the appropriate media in flat bottom 96-well culture plates in quadruplicate, which reached about 80% confluency in 48 hrs. Ten microliters containing 4-6 x 10^6^ of the four probiotic bacteria were added to each well and allowed to incubate overnight at 37 °C in room air containing about 18% oxygen.

Viability assay

The XTT assay (Biotium, Fremont, CA, USA) was used to evaluate cell viability at the end of culture experiments. XTT is a colorimetric detection assay utilizing tetrazolium dye to measure cell viability by enzymatic activity in the mitochondria of living cells. After the appropriate incubation time, 100 μl of the culture media from each well was removed for cytokine and chemokine analysis leaving 100 μl, to which 25 μl of the XTT reagent was added. The XTT plates were incubated at 37 °C for 120 minutes and the absorbance was recorded using a Tecan Genios plate reader (Mannedorf, Switzerland) that detects the absorption maximum (492 nm) of XTT.

One hundred microliters of PBMC culture supernatants (absent of cells) of the quadruplicate wells were combined for a total of 400 μl and frozen. The cell culture supernatants were sent on dry ice to Quansys Bioscience (Logan, Utah, USA) for the determination of cytokines and chemokine concentrations using an enzyme-linked immunosorbent assay (ELISA) chemiluminescent immunoassay.

Cytokines

Pro-inflammatory cytokines include interleukin-6 (IL-6), interleukin-1β (IL-1β), granulocyte-macrophage colony-stimulating factor (GMCSF), and tumor necrosis factor-alpha (TNFα). Interleukin-8 (IL-8) and monocyte chemoattractant protein (MCP-1) are chemokines that attract innate immune cells, especially granulocytes to areas of inflammation. The 15-cytokine multiplex assay done at Quansys measures all cytokines at the same time by highly sensitive chemiluminescence.

Statistical analysis

One-way analysis of variance (ANOVA) followed by Dunnett’s multiple comparisons test was performed using GraphPad Prism version 10.0.0 for Mac (GraphPad Software, Boston, MA, USA, www.graphpad.com).

## Results

It was our decision to study cellular viability and cytokine production after incubating PBMC with probiotic mixtures of anaerobic bacteria. With 70-80% of immune cells residing in the gut, it is important to understand the intricate interactions between the local microbiota and immune cells. First, there was no sign of contamination or bacterial overgrowth in any of the PBMC or HIEC-6 cultures. Adding the four probiotic samples containing different anaerobic bacteria stimulated the PBMC to produce up to a couple of thousand picograms/ml of the various cytokines/chemokines (Table 3).

**Table 3:** Cytokine determinations (pg/ml) in PBMC supernatant (bacteria only) Measurements of cytokines and chemokines in PBMC. Control: PBMC only in media. The addition of the four probiotic bacteria mixtures to PBMC stimulated cytokine production. Quadruplicate culture wells contained 2x10^5^ PBMC/well in 200 µl of CLT media and 10 µl of bacteria (NB 6.2x10^6^, MB 6.0x10^6^, M/F 4.1x10^6^ and IB 5.8x10^6^). PBMC: peripheral blood mononuclear cell; GMCSF: granulocyte-macrophage colony-stimulating factor; IL: interleukin; TNFα: tumor necrosis factor-alpha; MCP-1: monocyte chemoattractant protein-1

	GMCSF	IL-1β	IL-6	TNFα	IL-8	MCP-1
Control	6.5±0.8	15±0.9	9.7±0.6	27±0.6	509±28	80±11
NB	676±78	84±4.6 **	726±32 **	657±23 ***	6530±421 **	86±0.9
MB	458±39	57±4.6 *	1172±50 **	470±34 **	7335±128 ***	555±25 **
M/F	1844±91 *	283±6.8 ***	1851±47 ***	796±22 ***	5442±398 **	168±3.7 **
IB	1033±31 *	97±4.2 **	1514±52 ***	681±28 **	6208±238 **	427±1.4 ***

Adding PHA to the PBMC cultures stimulated the cells to produce higher amounts of the various cytokines and combining PHA and anaerobic bacteria produced even higher levels of various cytokines. However, the PHA bacteria combination induced significantly lower levels of MCP-1 compared to PHA alone (Table 4). Adding bacteria to the HIEC-6 cultures did not induce the production of pro-inflammatory cytokines or chemokines (data not shown).

**Table 4:** Cytokine determinations (pg/ml) in PBMC supernatants (PHA plus bacteria) PHA controls contained 10 µg/ml of PHA without bacteria. The four probiotic bacteria samples containing 10 mg/ml PHA were compared to PHA-only cultures for statistical purposes; the mean and standard deviation of each parameter are shown. Samples that induced inhibition of the chemokines IL-8 and MCP-1 production are in italics. One-way ANOVA followed by Dunnett’s multiple comparisons test was performed. ****p < 0.0001, ***p < 0.001, **p < 0.01 compared to the PHA control. PBMC: peripheral blood mononuclear cell; PHA: phytohemagglutinin; IL: interleukin; MCP-1: monocyte chemoattractant protein-1; ANOVA: analysis of variance

	GMCSF	IL-1β	IL-6	TNFα	IL-8	MCP-1
PHA Control	6800±230	859±22	20274±1400	7103±88	68016±720	11406±104
NB + PHA	12854±262 **	4212±163 **	25651±3891	7162±112	72432±3152	4034±108 ***
MB + PHA	8649±36 *	4347±11 ****	22986±834	8181±156 *	96049±2469 **	5661±127 ***
M/F + PHA	12440±79 **	5471±52 ***	24970±946 *	10092±226 **	62157±917 **	4245±12 ***
IB + PHA	14375±282 **	7182±156 ***	36508±15410	9349±225 *	77977±4066	3193±22 ***

## Discussion

**Figure 1:**
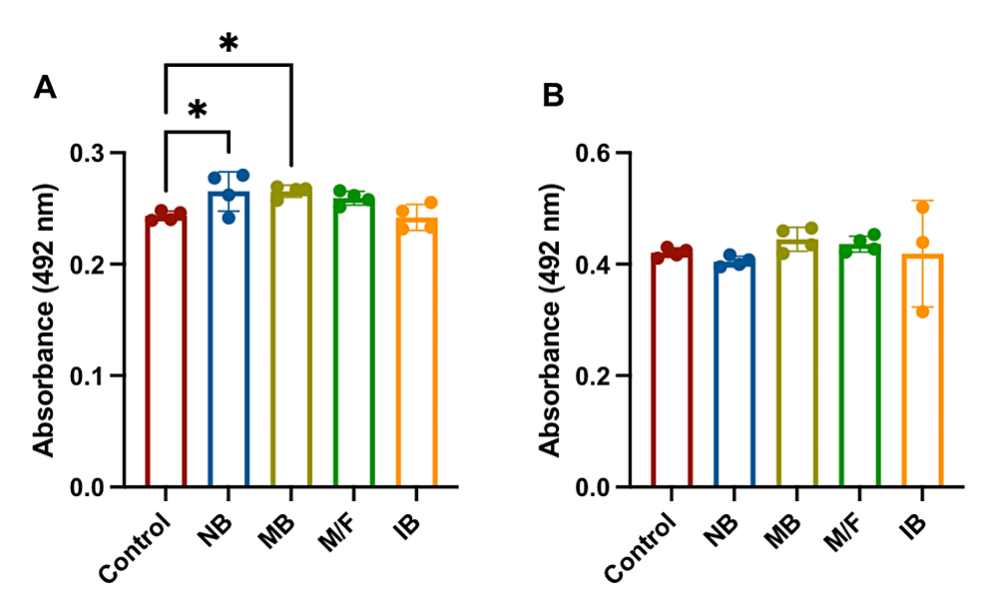
Cell viability A) XTT assay of PBMC after overnight culture. Control samples did not contain any bacteria. The cell viability of quadruplicate wells contained 2 x10^5^ PBMC/well in 200 µl of CLT media and 10 µml of bacteria (NB 6.2x10^6^, MB 6.0x10^6^, M/F 4.1x10^6^ and IB 5.8x10^6^) were examined after overnight culture. B) XTT assay of HIEC-6 after culture. Control samples did not contain any bacteria. Cell viability of cultures to which 10 µl of bacteria (NB 6.2x10^6^, MB 6.0x10^6^, M/F 4.1x10^6^, and IB 5.8x10^6^) were added to quadruplicate wells and examined after overnight culture. *p < 0.05 compared to the control samples. PBMC: peripheral blood mononuclear cell

There was a minor difference in cell viability in the control cultures with no bacteria compared to the cultures with four probiotic mixtures as measured by the XTT assay (Figures 1A, 1B). This is not surprising, as bacteria have molecules on their surfaces that bind to pattern recognition receptors (PRR) on immune cells even if the bacteria are dead.

These interactions of PRR on host cells and pathogen-associated molecular patterns (PAMPs) on bacteria are well-recognized and PAMPs exist in non-pathogenic bacteria [25]. PBMCs have toll-like receptors (TLRs) and C-type lectin receptors, which recognize bacterial PAMPs. Additionally, it is well known that cytokines are produced by microbe-immune cell interactions [26]. Cytokines are important chemical messengers that affect many cellular functions such as inflammation, cellular activation, and cellular proliferation [27].

**Figure 2:**
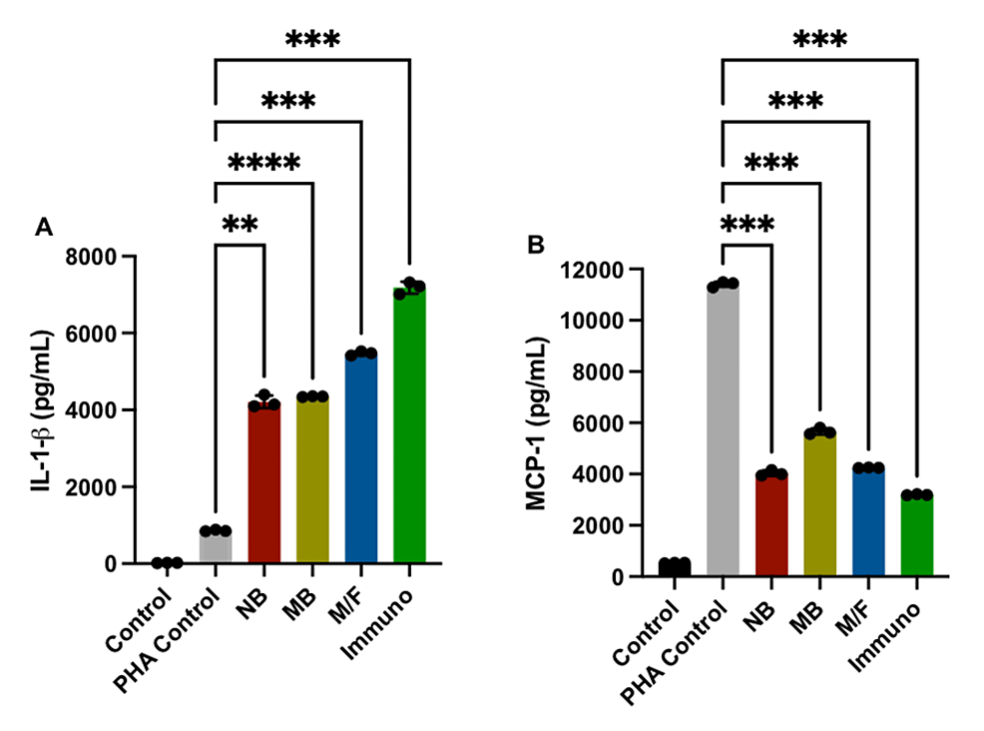
The production of cytokines and chemokines in PBMC cultures by the combination of bacteria and PHA. A) IL-1β production is significantly stimulated and B) MCP-1 production is significantly inhibited. ****p < 0.0001, ***p < 0.001, **p < 0.01 compared to the PHA control. PBMC: peripheral blood mononuclear cell; PHA: phytohemagglutinin

The innate immune system has several defense mechanisms to detect and respond to invading microorganisms [26]. One important response is the induction of inflammation to eliminate the invading microorganism. However, one must remember that there are trillions of bacteria living in our bodies. The coexistence of the countless strains of bacteria living in us cannot elicit full-blown inflammation like certain pathogenic infections. Therefore, inflammation must be carefully controlled or regulated to prevent excess tissue damage [28,29]. The inflammatory cytokine (IL-1β) production was significantly higher in PHA plus bacteria compared to the PHA control (Figure 2A), whereas the combination of PHA and bacteria significantly decreased the chemokine MCP-1 (Figure 2B).

Although this research strongly suggests that anaerobic bacteria do not overgrow PBMC when cultured overnight in media containing oxygen, there are many unanswered questions. For example, in the same experiment, the mixture of the four anaerobic bacteria with PHA resulted in 4-7-fold production of IL-1β (Figure 1A) and conversely a 2-4-fold decrease in the production of MCP-1 (Figure 2B) over PHA alone.

It was decided to look at another non-immune cell to determine if the anaerobic bacteria affected growth patterns. The epithelial cell (HIEC-6) is a normal cell line that resides in the vicinity of the bulk of intestinal bacteria. The HIEC-6 growth patterns with the four anaerobic probiotic bacteria are not significantly different from control cultures without bacteria (Figure 1B) and no evidence of contamination was noted under careful microscopic examination. This is further evidence that the anaerobic bacteria do not rapidly expand in oxygen-containing media.

The ability to examine anaerobic bacteria in culture with living cells in overnight culture suggests numerous experimental possibilities. The PBMC used in these experiments contained a mixture of white cells, and it is unclear which cells are responding to the bacteria. Purified monocytes, T-cells, B-cells, dendritic cells, etc. should be examined to determine anaerobic bacterial effects.

Also, PBMC or white cells from individuals with specific diseases could be examined for cytokine responses. Single anaerobic bacteria should be examined to determine specific cytokine effects. Culture experiments longer than overnight may show differences and there may be stimulation of other cytokines or growth factors not measured in our current assays. Gene expression experiments could be useful, in certain experiments, to determine bacterial effects on a particular cell line.

In our hands, the mixing of anaerobic bacteria with human cells in culture showed some interesting results. Limitations in new areas of research become apparent when different laboratories repeat similar experiments. Our research presented here evaluated four mixtures of probiotics of anaerobic bacteria. The first limitation of this work is there is the limited amount of data generated from this novel approach of combining anaerobic bacteria with cells in culture. The second limitation is we only looked at a small number of cytokines. Thirdly, we do not know which individual bacteria in the four mixtures is causing the cytokine effect, suggesting that cell culture experiments should be done using individual anaerobic bacteria. The last limitation is we only examined a small number of bacteria compared to the thousands of anaerobic bacteria that exist in the intestines.

## Conclusions

The data presented here clearly suggests that anaerobic bacteria do not grow rapidly in oxygen-containing conditions in the PBMC cell culture experiments. The presence of anaerobic bacteria in PBMC cultures stimulates a weak pro-inflammatory response in several cytokines. The addition of PHA and PHA plus anaerobic bacteria results in a more robust cytokine response. In the same experiments, PHA plus anaerobic bacteria significantly inhibits the chemokine MCP-1 response. The growth patterns of the normal cell line HIEC-6 are not strongly affected by the four anaerobic bacteria mixtures, nor was there a robust cytokine response.
